# Benchmarking the American Society of Breast Surgeon Member Performance for More Than a Million Quality Measure-Patient Encounters

**DOI:** 10.1245/s10434-017-6257-9

**Published:** 2017-11-22

**Authors:** Jeffrey Landercasper, Oluwadamilola M. Fayanju, Lisa Bailey, Tiffany S. Berry, Andrew J. Borgert, Robert Buras, Steven L. Chen, Amy C. Degnim, Joshua Froman, Jennifer Gass, Caprice Greenberg, Starr Koslow Mautner, Helen Krontiras, Luis D. Ramirez, Michelle Sowden, Barbara Wexelman, Lee Wilke, Roshni Rao

**Affiliations:** 1 Gundersen Medical Foundation, La Crosse, WI USA; 2 Duke University, Durham, NC USA; 3 Bay Area Breast Surgeons, Inc, Oakland, CA USA; 4 Norton Healthcare, Louisville, KY USA; 5 Anne Arundel Medical Center, Annapolis, MD USA; 6 OasisMD, San Diego, CA USA; 7 Mayo Clinic, Rochester, MN USA; 8 Mayo Clinic Health System, Owatonna, MN USA; 9 Women and Infants Hospital, Providence, RI USA; 10 University of Wisconsin School of Public Health and Medicine, Madison, WI USA; 11 Miami Cancer Institute, Baptist Health South Florida, Miami, FL USA; 12 University of Alabama at Birmingham, Birmingham, AL USA; 13 University of Vermont Medical Center, Burlington, VT USA; 14 Trihealth Cancer Institute, Cincinnati, OH USA; 15 Columbia University Medical Center, New York, NY USA

## Abstract

**Background:**

Nine breast cancer quality measures (QM) were selected by the American Society of Breast Surgeons (ASBrS) for the Centers for Medicare and Medicaid Services (CMS) Quality Payment Programs (QPP) and other performance improvement programs. We report member performance.

**Study Design:**

Surgeons entered QM data into an electronic registry. For each QM, aggregate “performance met” (PM) was reported (median, range and percentiles) and benchmarks (target goals) were calculated by CMS methodology, specifically, the Achievable Benchmark of Care™ (ABC) method.

**Results:**

A total of 1,286,011 QM encounters were captured from 2011–2015. For 7 QM, first and last PM rates were as follows: (1) needle biopsy (95.8, 98.5%), (2) specimen imaging (97.9, 98.8%), (3) specimen orientation (98.5, 98.3%), (4) sentinel node use (95.1, 93.4%), (5) antibiotic selection (98.0, 99.4%), (6) antibiotic duration (99.0, 99.8%), and (7) no surgical site infection (98.8, 98.9%); all *p* values < 0.001 for trends. Variability and reasons for noncompliance by surgeon for each QM were identified. The CMS-calculated target goals (ABC™ benchmarks) for PM for 6 QM were 100%, suggesting that not meeting performance is a “never should occur” event.

**Conclusions:**

Surgeons self-reported a large number of specialty-specific patient-measure encounters into a registry for self-assessment and participation in QPP. Despite high levels of performance demonstrated initially in 2011 with minimal subsequent change, the ASBrS concluded “perfect” performance was not a realistic goal for QPP. Thus, after review of our normative performance data, the ASBrS recommended different benchmarks than CMS for each QM.

Gaps in the quality of healthcare exist in the United States.^1^–^21^ As a consequence, measures of quality have been developed and initiatives launched to provide peer comparisons as a method for quality improvement.^4^,^9^,^10^,^14^–^17^,^20^,^22^–^35^ Building on these efforts, payers of healthcare introduced public reporting and “pay for performance” programs.^36^

Recognizing the need to search for gaps in care, the American Society of Breast Surgeons (ASBrS) built a patient registry called Mastery of Breast Surgery^SM^ (Mastery) and developed quality measures (QM) to audit.^24^ In Mastery, surgeons can view their own performance and immediately compare themselves to other surgeons after they enter data. As early as 2009, nearly 700 member surgeons of the ASBrS demonstrated their commitment to QM reporting by entering data on 3 QM for each of 28,000 breast cancer cases.^13^ We updated the results of the ASBrS measurement program for those QM accepted by the Centers for Medicare and Medicaid Services (CMS) for their quality payment programs (QPP) (Table 1).^36^ Our purpose was to provide transparency of member performance, investigate for variability of care, and describe how this information was used to develop quality targets (benchmarks). To our knowledge, we report the largest sample of breast surgeon-entered QM encounters assembled to date.

**Table 1:** American Society of Breast Surgeons quality measures (QM)^44^

QM title	QM name	QM numerator	QM denominator
Needle biopsy	PQRS measure #263: Preoperative diagnosis of breast cancer	Number of patients age 18 and older undergoing breast cancer operations, who had breast cancer diagnosed preoperatively by a minimally invasive biopsy	Number of patients age 18 years and older on date of encounter undergoing breast cancer operations
Image confirmation	PQRS measure #262: Image confirmation of successful excision of image-localized breast lesion	Patients undergoing excisional biopsy or partial mastectomy of a nonpalpable lesion whose excised breast tissue was evaluated by imaging intraoperatively to confirm successful inclusion of targeted lesion	Number of patients aged 18 years and older on date of encounter with nonpalpable, image-detected breast lesion requiring localization of lesion for targeted resection
Sentinel node	PQRS measure #264: Sentinel lymph node biopsy for invasive breast cancer	Patients who undergo a sentinel lymph node biopsy procedure	Patients aged 18 and older with clinically node-negative stage 1 and 2 primary invasive breast cancer
Hereditary assessment	ASBrS 1: Surgeon assessment for hereditary cause of breast cancer	Number of newly diagnosed invasive and ductal carcinoma in situ (DCIS) breast cancer patients seen by surgeon that undergo risk assessment for a hereditary cause of breast cancer	Number of newly diagnosed invasive and DCIS breast cancer patients seen by surgeon
Surgical site infection^a^	ASBrS 2: Surgical site infection and cellulitis after breast and/or axillary surgery	Number of patients aged 18 and over who developed an SSI or cellulitis within 30 days of undergoing a breast and/or an axillary operation	Number of patients aged 18 years and older on date of encounter undergoing a breast and/or axillary operation
Specimen orientation	ASBrS 3: Specimen orientation for partial mastectomy or excisional breast biopsy	Number of patients age 18 and older undergoing a therapeutic breast surgical procedure considered an initial partial mastectomy or “lumpectomy” for a diagnosed cancer or an excisional biopsy for a lesion that is not clearly benign based on previous biopsy or clinical and radiographic criteria with surgical specimens properly oriented for pathologic analysis such that six margins can be identified	Number of patients age 18 and older undergoing a therapeutic breast surgical procedure considered an initial partial mastectomy or “lumpectomy” for a diagnosis of cancer or an excisional biopsy for a lesion that is not clearly benign based on previous biopsy or clinical and radiographic criteria
Antibiotic choice	ASBrS 5: Perioperative care: selection of prophylactic antibiotics: first- *or* second-generation cephalosporin (modified for breast from PQRS measure #21)	Surgical patients aged 18 years and older undergoing procedures with indications for a first- *or* second-generation cephalosporin prophylactic antibiotic, who had an order for a first- *or* second-generation cephalosporin for antimicrobial prophylaxis	All surgical patients aged 18 years and older undergoing procedures with the indications for a first OR second generation cephalosporin prophylactic antibiotic
Antibiotic duration	ASBrS 6: Perioperative care: discontinuation of prophylactic parenteral antibiotics (modified for breast from PQRS measure #22)	Noncardiac surgical patients who have an order for discontinuation of prophylactic parenteral antibiotics within 24 h of surgical end time	All noncardiac surgical patients aged 18 years and older undergoing procedures with the indications for prophylactic parenteral antibiotics *and* who received a prophylactic parenteral antibiotic
Mastectomy reoperation	Unplanned 30 day reoperation rate after mastectomy	Patients undergoing mastectomy who do not require an unplanned secondary breast or axillary operation within 30 days of the initial procedure	Patients undergoing unilateral or bilateral mastectomy as their initial procedure for breast cancer or prophylaxis

^a^Mastectomy with reconstruction is included

## Methods

De-identified QM data were obtained from the ASBrS for the years 2011–2015. Due to de-identification, the Institutional Review Board of the Gundersen Health System deemed the study was not human subject research; the need for formal IRB approval was waived.

### CMS Rules and Formulas

All QM must be *specified with inclusion, exclusion, and exception criteria* (Table 1).^36^,^37^

Using “performance met” (PM) and “performance not met” (PNM) for each QM, the formula for performance rate (PR) was as follows: PR = [PM]/[PM + PNM]. Patients with *exceptions* are included in the PR only if there was PM. *Excluded* patients are never included in the PR. For example, patients undergoing lumpectomy are excluded from the mastectomy reoperation QM.

For calculating the total number of surgeon–patient-measure encounters captured in Mastery, we summed the total reports for each individual QM for all study years and all providers who entered data. Statistical Analysis Software, version 9.3 (SAS Institute Inc., Cary, NC) was used to report performance.

Benchmarks for performance for each QM were calculated by the Achievable Benchmark of Care™ (ABC) methodology recommended by CMS.^38^,^39^ ABC benchmarks were reviewed by the ASBrS Board of Directors in person on January 22, 2016. By the ABC method, calculated benchmarks for six QM were 100% performance met. Thus, for these measures, performance not met became a defacto “never-should occur event.” As a result, the Patient Safety and Quality (PSQ) and the executive committees recommended different benchmarks to be based on our member normative performance data and society expert opinion. This methodology of setting a target goal for passing a test has been termed a modified Angoff approach by educators and is similar to the process used by the European Society of Breast Cancer Specialists (EUSOMA).^28^,^40^–^43^ To assess annual trends in performance, the Cochrane Armitage test was used.

### Society Actions

The ASBrS performed an annual review of participating member performance for the QM captured in Mastery. The results were presented to their Board of Directors by the PSQ Committee. Initiatives to address quality concerns were then discussed or planned.

## Results

### Encounters Captured

A total of 1,286,011 unique provider-patient-measure encounters were captured in Mastery during 2011–2015 for 9 QCDR QM.^44^ Encounters varied by QM from 275,619 for the specimen orientation QM to 2680 for a recently introduced hereditary risk QM (Table 2). The number of encounters differed by QM due to its eligibility requirements and the time point when it was first available for reporting. The dropout rate of surgeons who did not enter any encounters for the last reporting year (2015) but who had entered data in prior years was 43% (354/832).

**Table 2:** Quality measure “performance met” and benchmarks 2011–2015

QM name	No. reporting surgeons	Society aggregate performance met % (N/D)	Range (%)	25th percentile (%)	50th percentile (%)	75th percentile (%)	90th percentile (%)	Benchmark^a^	
								CMS ABC^TM^	ASBrS recommended
Needle biopsy	476	97.5% (230,187/236,167)	(14–100)	93	99	100	100	100%	90%
Specimen orientation	473	98.6% (271,876/275,619)	(11–100)	98	100	100	100	100%	95%
Specimen imaging confirmation	438	98.5% (145,061/147,228)	(5–100)	93	100	100	100	100%	95%
Sentinel node appropriate use in clinical node negative patients	460	94.4% (108,102/114,455)	(1–100)	89	98	100	100	100%	90%
No unplanned reoperation after mastectomy	406	98.6% (22,879/23,204)	(12–100)	94	100	100	100	100%	< 10%
Antibiotic selection of first-generation cephalosporin	460	98.9% (172,555/174,434)	(3–100)	94	100	100	100	NA^b^	NA^b^
Antibiotic stopped after 24 h	450	99.3% (169,082/170,261)	(8–100)	97	100	100	100	NA^b^	NA^b^
Hereditary risk	143	86.3% (2314/2680)	(29–100)	64	75	88	97	98%	90%
No post-op surgical site infection	547	98.6% (139,956/141,963)	(1–100)	94	99	100	100	100%	< 6%

Numerator is “performance met.” Denominator is “performance met” + “performance not met”

^a^CMS Benchmark was derived from ABC™ formula; the ASBrS Benchmarks were determined after calculating and not endorsing the ABC™ benchmarks. The ASBrS Benchmarks were based on the observed normative performance data in this study and expert opinion of the ASBrS Quality and Executive Committees

^b^Not applicable because measures are retired

### Performance

**Fig. 1:**
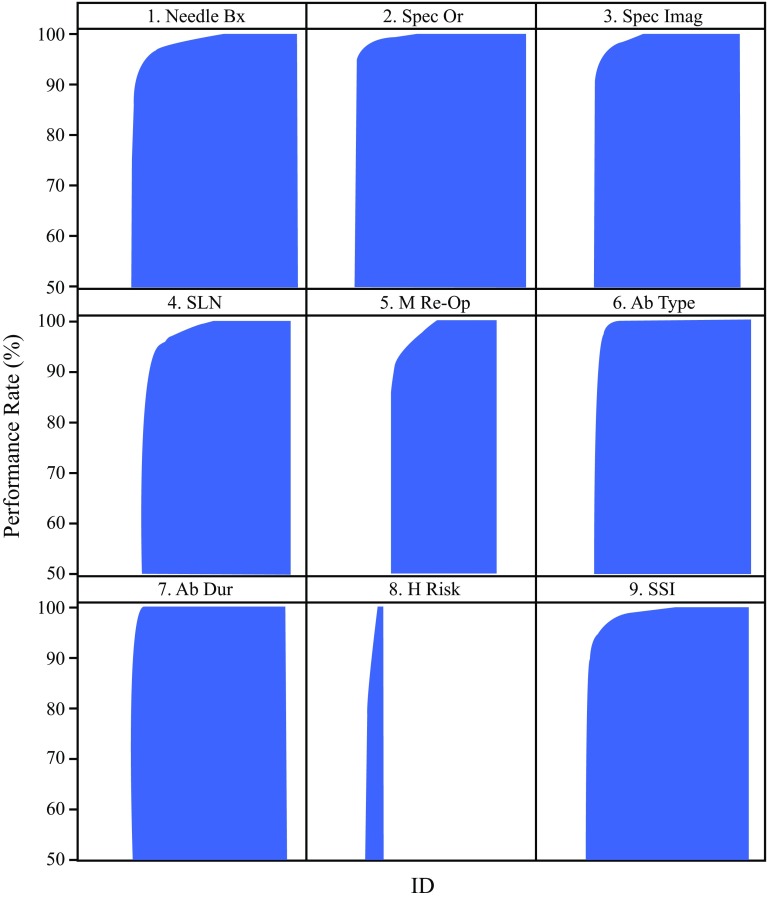
Histograms of individual surgeons and their performance. *X*-axis is individual surgeon de-identified ID numbers; *Y*-axis is performance rate from 50 to 100% “performance met”

Performance and benchmarks are shown in Table 2. Performance variability and trends are shown in Fig. 1 and Table 3. The initial and last performance met rates for seven QM from 2011–2015 were as follows: needle biopsy (NB) (95.8, 98.5%), specimen imaging (SI) (97.9, 98.8%), antibiotic selection (AS) (98.0, 99.4%), antibiotic duration (AD) (99.0, 99.8%), no surgical site infection (NSSI) (98.8, 98.9%), specimen orientation (SO) (98.5, 98.3%), and sentinel node use (SN) (95.1, 93.4%); all *p* values < 0.001, indicating significant improvement in the first five QM and worsening in the last two. The performance of three QM available before 2011, reported by Clifford et al., compared with 2015, demonstrated improvement as follows: needle biopsy (73–98.5%), specimen orientation (84–98.3%), and specimen imaging (47–98.8%); all *p* values < 0.001.^13^

**Table 3:** Annual trends of performance

Quality measure	Aggregate performance (%)	*p* value	Status
Needle biopsy			
2011	95.8	< 0.0001	Improved
2012	97.2		
2013	97.3		
2014	97.3		
2015	98.5		
Specimen orientation			
2011	98.5	< 0.0001	Decreased
2012	98.7		
2013	99.0		
2014	99.0		
2015	98.3		
Specimen imaging confirmation			
2011	97.9	< 0.0001	Improved
2012	98.6		
2013	98.6		
2014	98.3		
2015	98.8		
Sentinel node appropriate use in clinical node-negative patients			
2011	95.1	< 0.0001	Decreased
2012	94.6		
2013	94.6		
2014	95.9		
2015	93.4		
Antibiotic selection of first-generation cephalosporin			
2011	98.0	< 0.0001	Improved
2012	99.1		
2013	98.9		
2014	98.5		
2015	99.4		
Antibiotic stopped after 24 h			
2011	99.0	< 0.0001	Improved
2012	98.9		
2013	98.8		
2014	99.3		
2015	99.8		
Post-op surgical site infection			
2011	98.8	< 0.0001	Improved
2012	98.5		
2013	97.5		
2014	98.0		
2015	98.9		

The hereditary assessment and the unplanned reoperation after mastectomy QM not included in trending analysis, because they are new measures

The most common reasons for performance not met (PNM) by QM were “patient refusal” for NB (0.6%, 583/105,541), “fragmented tissue” for SO (0.6%, 599/105,186), “imaging not available” for SI (0.04%, 41/95,534), “attempted, not successful” for SN (0.6%, 627/99,172), “no reason given” for AS (0.4%, 419/97,206), “no reason given” for AD (0.1%, 136/96,583), “infection” for NSSI (1.4%, 1987/141,963), and “bleeding” for mastectomy reoperation (0.2%, 152/73,886). Other reasons for PNM and exceptions for each QM are in Table 4.

**Table 4:** Quality measurement “exceptions” and “performance not met (PNM)”

Quality measurement	Most common exceptions N/D (%)		Most common “PNM” (number of patients)
Needle biopsy	Prophylactic mastectomy	8264/105,541 (7.8%)	Patient refused needle biopsy (583) Needle biopsy not available in my community (1)
	Lesion too close to skin, implant, chest wall, etc.	4503/105,541 (4.3%)	
	Clinical and imaging findings consistent with a benign lesion	1814/105,541 (1.7%)	
Specimen orientation	Clinical and imaging findings consistent with a benign lesion	3644/105,186 (3.5%)	Tissue fragmented during removal (599) Specimen handling precluded orientation (45)
	Orientation specimen would add no value (recurrent disease, etc.)	440/105,186 (0.4%)	
Specimen imaging confirmation	Target verified on intraoperative inspection or pathology	1022/95,534 (1.1%)	Appropriate imaging modality was not available for confirmation (41)
	MRI or PEM wire localization without marker placement	16/95,534 (0.02%)	
Sentinel node appropriate use in clinical node-negative patients	Patient with significant age, comorbidities, or limited life expectancy and favorable tumor; adjuvant systemic treatment unlikely to change	4257/99,172 (4.3%)	Attempted but not successful (627)
	SLN or ALND procedure previously performed	4044/99,172 (4.1%)	
	Recurrent invasive cancer	223/99,172 (0.2%)	
Unplanned reoperation after mastectomy	From plastic surgeon attribution to include transfer of primary care	36/73,886 (0.05%)	Bleeding requiring exploration (152) Margin close or positive (41)
	Reconstructive flap necrosis	29/73,886 (0.04%)	
	From pathologist attribution	15/73,886 (0.02%)	
Antibiotic selection of first-generation cephalosporin	Cefazolin or cefuroxime NOT ordered- Allergy to penicillin or cephalosporin	7889/97,206 (8.1%)	Cefazolin or cefuroxime NOT ordered, no reason specified (419)
	Cefazolin or cefuroxime NOT ordered for medical reason	236/97,206 (0.2%)	
Antibiotic stopped after 24 h	Antibiotic NOT discontinued – ordered by plastic surgeon for expander or implant insertion	5898/96,583 (6.1%)	No reason specified, antibiotic NOT discontinued (or ordered to be) within 24 h (and given within 4 h prior to incision or intraoperatively) (136)
	For medical reasons, antibiotic NOT discontinued (or ordered to be) within 24 h (and given within 4 h prior to incision or intraoperatively)	1417/96,583 (1.5%)	
	Antibiotic NOT discontinued—ordered by plastic surgeon for autologous flap	842/96,583 (0.9%)	
Hereditary risk	Genetic testing denied by insurance	100/7047 (1.4%)	Genetic testing not ordered, no reason specified (166) Patient refused genetic testing (117)
Post-op surgical site infection	No “exceptions”		Cellulitis (435) Superficial SSI (310) Deep SSI (116)

Exceptions mean the surgeon is not penalized in their performance rate for not meeting performance because the reason for PNM is justifiably not related to quality, as determined by the American Society of Breast Surgeons

### Benchmarks

With the CMS ABC formula, the benchmarks were 100% performance met for every QM except for the hereditary risk measure, which was 98%. In contrast, the ASBrS-recommended benchmarks were as follows: needle biopsy (90%), specimen orientation (95%), specimen imaging (95%), sentinel node use (90%), mastectomy reoperation rate (< 10%), hereditary risk assessment (90%), and surgical infection (< 6%; Table 2). Benchmarks for the two antibiotic QM were not established, because they have been discontinued.

## Discussion

### Background

In 2008, the ASBrS launched its Mastery program for breast surgeons to document the quality of their clinical performance.^13^ After modified Delphi ranking, 9 of 144 breast surgical QM were chosen for ASBrS member self-assessment, benchmarking and CMS QPP.^44^ Program developers and ranking participants had diversity of practice location and type to include nonspecialty breast surgeons.

### Participation

By 2017, spurred by landmark legislation and the need to improve quality, nearly 70 organizations developed patient registries for clinicians to report more than 300 QM to CMS.^36^,^45^–^48^ Our registry which started much earlier has already successfully captured over one million unique patient-measure encounters and provided real-time benchmarking.

### Performance

There was a high rate of performance for eight of our nine measures. For these eight measures, compliance was met in more than 94% of patient encounters. Notable examples include the high rate of preoperative diagnosis of breast cancer made by a needle biopsy (97.5%) and the low rates of surgical site infection and unplanned reoperation after mastectomy: both less than 2%. This level of performance exceeded most historical reports.^18^,^49^–^51^ The QM with the lowest aggregate performance was “documentation of surgeon hereditary assessment of a newly diagnosed breast cancer patient” at 86%. Overall performance for the other eight QM was excellent. However, we recognize that disparities of care may still be present. During the last 6 years of measurement, there were statistically significant changes in performance for all measures. Despite both the upward- and downward- trending changes, the absolute differences by year were small, all less than 3%, which raises the question of these changes’ clinical significance. Because the performance level of surgeons reporting in Mastery is so high, it is possible these surgeons may be a self-selected group of high-performing surgeons. Supporting this concept, surgeons voluntarily reporting in a cardiac surgery registry, compared with nonparticipants, demonstrated better performance.^52^ Our findings of such high performance in the initial study years, followed by minimal annual change, is similar to a recent report from European breast centers.^53^ When this scenario occurs, there is concern that these QM may have “topped out,” resulting in less opportunity for future improvement. However, because the level of performance for nonparticipants in our program is unknown, we have not yet retired our QM; rather by continuing to support them, we are endorsing their importance inside and outside our society membership.

Although aggregate performance rates were high, variability of performance existed, best demonstrated by histograms (Fig. 1). Whenever variability coexists with evidence that high performance is achievable, there is opportunity to improve overall care.^54^

### When Performance is Not Met, What Can We Learn?

The most common reasons for not meeting performance for each measure are documented in Table 3. Even with high overall performance, there is value to identifying causes of measure noncompliance. Understanding causation affords opportunity to improve. For example, one reason for omission of a needle biopsy for diagnosing cancer was “needle biopsy not available in my community,” which represents a system and resource issue, rather than a surgeon-specific issue. Supporting solutions, the ASBrS has provided education and certification for both ultrasound-guided and stereotactic core needle biopsy.^55^ In another example, the second most common reason that patients underwent an unplanned reoperation after mastectomy was for a positive margin. Potentially, surgeons learning they are comparative outliers for margin involvement may reevaluate their care processes to better assess the cancer’s proximity to the mastectomy margins preoperatively.

If performance is not met for a QM due to a justifiable “nonquality” reason, then CMS defines this encounter as an “exception.” In such cases, the encounter did not penalize the surgeon, because it was not included in their performance rate. An exception to not meeting performance for achieving a cancer diagnosis by a needle biopsy occurred in 8264 patients undergoing prophylactic mastectomy and in 1814 patients having an imaging abnormality that was “too close” to an implant or the chest wall to permit safe needle biopsy. This granular level of information potentially aids improvement strategies. For example, in high-risk patients undergoing risk-reducing mastectomy, surgeons ought to pursue guideline concordant preoperative imaging to identify nonpalpable cancers, thereby improving both the needle-biopsy rate for cancer as well as reducing the mastectomy reoperation rate by excising sentinel nodes during the initial mastectomy in patients later found to have invasive cancer.

Capturing exceptions also allowed for accurate attribution assignments. For example, in our registry, a surgeon can attribute a reoperation after mastectomy to themselves, such as for axillary bleeding, or to the plastic surgeon for flap donor site bleeding.

### Benchmarking

*Benchmarking (profiling)* means that participants can compare their performance to others and is a method for quality improvement.^23^,^31^,^39^,^53^,^56^,^57^ Benchmarking programs differ. Navathe et al. recently summarized eight different design factors.^56^ Using this categorization, our program is identity-blind, reports textually (not graphically), encourages high-value care, discourages low-value care, compares an individual to a group, contains measures with both higher and lower levels of evidence supporting them, has a national scope, and to our knowledge has not resulted in any unintended adverse outcome.

The term *benchmark* means a point of reference. A benchmark may simply be an observation of results of contemporary care, perhaps when first described in a specific patient population.^39^,^58^ A benchmark also can be an organizational target goal, such as a zero percent infection rate, or a data-driven reference, reached when content experts scrutinize observed ranges of performance and subsequently endorse a specific percentile.^40^–^43^ In 2008, 24 breast cancer experts attended a workshop in Europe and established benchmarks for 17 quality indicators for breast centers, calling them *minimum standards* and *quality targets*.^28^,^53^ The establishment of a quality target is a known method for improving quality beyond the effect of peer comparison.^35^ Recognizing this concept, CMS requires that QCDR stewards determine ABC benchmarks for each of their QM.^38^ Conceptually, the ASBrS Board of Directors agreed that benchmarks can be catalysts for improvement. After application of the ABC formula, the CMS benchmarks for six of our QM were 100% “performance met.” After review, the ASBrS Board concluded that achieving perfection in every patient encounter was desirable but should not be considered the “standard of care”; nor should “performance not met” be considered a “never” event. As a result, the ASBrS Quality Committee and Board reviewed the member performance presented here, as well as relevant literature, then endorsed different benchmarks, that reflected high-quality and clinically achievable care (Table 2).

### Was our Quality Program the Driver of Observed Improvements in Performance?

For the first three QM that measured needle biopsy, specimen imaging, and specimen orientation rates in our program before 2011, there was marked improvement compared with 2011–2015.^13^ Overall, there was significant improvement for seven of our nine QM from 2011 to 2015. Whether this improvement was directly related to our measurement and benchmarking, and the natural consequence of measurement driving improvement, cannot be conclusively determined given multiple competing reasons that could explain improvement. These potential confounders include some changes in QM specifications over time, as well as our own educational programs and scholarly publications within and outside our Society.^53^

### Program and Study Strengths and Limitations

The strengths and limitations of the ASBrS Mastery patient registry have been described elsewhere.^44^ Strengths include large sample sizes, immediate peer comparison, and appropriate attribution assignments.^44^ In addition, our registry is flexible in terms of its ability to capture additional data fields after appropriate vetting by the society and in its ability to output data across a number of domains. While it was initially developed for quality measurement, it also has been used for clinical outcomes research.^13^,^59^–^62^

Limitations are recognized.^44^ A selection bias is possible because the surgeons who self-select to participate may be “above average.” They may share certain characteristics, such as a focus on quality and safety, better resources or a different case-mix compared to nonparticipating surgeons. If so, our results may not be reproducible in other settings. Due to this concern, the ASBrS Board agreed to offer participation to non-ASBrS members for pilot studies. Other limitations include an unknown rate of nonconsecutive case entry and an unknown rate of surgeon dropout due to their perception of poor performance. In addition, most of our QM are process rather than outcome measures, and we are not providing risk-adjusted comparisons. As a result, investigations are underway to identify the interactions between patient, surgeon, and facility characteristics that affect our measured outcomes. Lastly, formal reliability testing of our measures and advanced analytic tools to disentangle each surgeon’s intrinsic performance from their supporting institution have not been performed.^63^,^64^

## Conclusions

The ASBrS successfully constructed an electronic patient registry and then engaged breast surgeons to capture more than a million organ-specific QM encounters, providing proof of surgeons’ commitment to self-assessment as well as evidence of our societies’ compliance with a mission “continually to improve the practice of breast surgery.”^65^ Functionality was provided for surgeon profiling, program data were used to establish quality targets and a service was provided to surgeon members allowing them to participate in CMS incentivized reimbursement programs. Much work remains to include more advanced analytic methods for benchmarking and to decide when to retire existing measures that may have “topped out.” For now, we encourage all surgeons not participating in our program to compare their personal performance to our benchmarks. In addition, we are currently searching for inequities and disparities of care by surgeon and patient characteristics.

## References

[1] Rosenberg BL, Kellar JA, Labno A (2016). Quantifying geographic variation in health care outcomes in the United States before and after risk-adjustment. PLoS One.

[2] Levine D, Linder J, Landon B (2016). The quality of outpatient care delivered to adults in the United States, 2002 to 2013. JAMA Intern Med..

[3] Horwitz RI (2016). Equity in cancer care and outcomes of treatment: a different type of cancer moonshot. JAMA.

[4] American Society of Clinical Oncology (2015). The state of cancer care in America™, 2015: a report by the American Society of Clinical Oncologists. J Oncol Pract..

[5] Kohn LT, Corrigan J, Donaldson MS. To err is human: building a safer health system. Institute of Medicine (US) Committee on Quality of Health Care in America. Washington (DC): National Academies Press (US); 2000. 25077248

[6] Institute of Medicine (US) Committee on Quality of Health Care in America. Crossing the quality chasm: a new health system for the 21st century. Washington (DC): National Academies Press (US); 2001. 25057539

[7] Levit LA, Balogh E, Nass SJ, Ganz PA (2013). Delivering high-quality cancer care: charting a new course for a system in crisis.

[8] Hewitt M, Simone JV, (eds). Ensuring Quality Cancer Care. Institute of Medicine (US) and National Research Council (US) National Cancer Policy Board. Washington (DC): National Academies Press (US); 1999. 25101447

[9] Davis K, Stremikis K, Schoen C, Squires D (2014) Mirror, mirror on the wall, 2014 update: how the U.S. health care system compares internationally. The Commonwealth Fund. http://www.commonwealthfund.org/publications/fund-reports/2014/jun/mirror-mirror. Accessed 8 December 2015.

[10] Goodney PR, Dzebisashvili N, Goodman DC, Bronner KK (2015) Variation in the care of surgical conditions. The Dartmouth Institute. http://www.dartmouthatlas.org/downloads/atlases/Surgical_Atlas_2014.pdf. Accessed 8 Dec 2015. 36534748

[11] Balogh EP, Miller BT, Ball JR, eds. Improving diagnosis in health care. Committee on Diagnostic Error in Health Care; Board on Health Care Services; Institute of Medicine; The National Academies of Sciences, Engineering, and Medicine. Washington (DC): The National Academies Press (US); 2015. 26803862

[12] Agency for Healthcare Research and Quality (2015). National Healthcare Quality & Disparities Reports. http://www.ahrq.gov/research/findings/nhqrdr/index.html Accessed 8 Dec 2015.

[13] Clifford EJ, De Vol EB, Pockaj BA (2010). Early results from a novel quality outcomes program: the American Society of Breast Surgeons’ Mastery of Breast Surgery. Ann Surg Oncol.

[14] Malin JL, Diamant AL, Leake B (2010). Quality of care for breast cancer for uninsured women in California under The Breast and Cervical Cancer Prevention Treatment Act. J Clin Oncol..

[15] Wilke LG, Ballman KV, McCall LM (2010). Adherence to the National Quality Forum (NQF) breast cancer measures within cancer clinical trials: a review from ACOSOG Z0010. Ann Surg Oncol..

[16] Warner ET, Tamimi RM, Hughes ME (2015). Racial and ethnic differences in breast cancer survival: mediating effect of tumor characteristics and sociodemographic and treatment factors. J Clin Oncol..

[17] Bekelman JE, Sylwestrzak G, Barron J (2014). Uptake and costs of hypofractionated vs conventional whole breast irradiation after breast conserving surgery in the United States, 2008-2013. JAMA.

[18] Silverstein M (2009). Where’s the outrage?. J Am Coll Surg..

[19] Hassett MJ, Neville BA, Weeks JC (2014). The relationship between quality, spending and outcomes among women with breast cancer. J Natl Cancer Inst.

[20] Greenberg CC, Lipsitz SR, Hughes ME (2011). Institutional variation in the surgical treatment of breast cancer: a study of the NCCN. Ann Surg.

[21] Kent EE, Mitchell SA, Castro KM (2015). Cancer care delivery research: building the evidence base to support practice change in community oncology. Clin Oncol.

[22] Cohen ME, Liu Y, Ko CY, Hall BL (2016). Improved surgical outcomes for ACS NSQIP hospitals over time: evaluation of hospital cohorts with up to 8 years of participation. Ann Surg.

[23] Edge SB (2014). Quality measurement in breast cancer. J Surg Oncol.

[24] The American Society of Breast Surgeons (2016). Mastery of Breast Surgery^SM^ Program. https://www.breastsurgeons.org/new_layout/programs/mastery/background.php. Accessed 10 June 2016.

[25] Agency for Healthcare Research and Quality (2017). The National Quality Measures Clearinghouse. http://www.qualitymeasures.ahrq.gov/search/search.aspx?term=breast. Accessed 13 June 2016. 10.1080/15360280802537332 21923316

[26] The National Quality Forum (2016) Endorsed Breast Cancer Quality Measures. http://www.qualityforum.org/QPS/QPSTool.aspx#qpsPageState=%7B%22TabType%22%3A1,%22TabContentType%22%3A1,%22SearchCriteriaForStandard%22%3A%7B%22TaxonomyIDs%22%3A%5B%5D,%22SelectedTypeAheadFilterOption%22%3A%7B%22ID%22%3A13875,%22FilterOptionLabel%22%3A%22breast%22,%22TypeOfTypeAheadFilterOption%22%3A1,%22TaxonomyId%22%3A0%7D,%22Keyword%22%3A%22breast%22,%22PageSize%22%3A%2225%22,%22OrderType%22%3A3,%22OrderBy%22%3A%22ASC%22,%22PageNo%22%3A1,%22IsExactMatch%22%3Afalse,%22QueryStringType%22%3A%22%22,%22ProjectActivityId%22%3A%220%22,%22FederalProgramYear%22%3A%220%22,%22FederalFiscalYear%22%3A%220%22,%22FilterTypes%22%3A0,%22EndorsementStatus%22%3A%22%22%7D,%22SearchCriteriaForForPortfolio%22%3A%7B%22Tags%22%3A%5B%5D,%22FilterTypes%22%3A0,%22PageStartIndex%22%3A1,%22PageEndIndex%22%3A25,%22PageNumber%22%3Anull,%22PageSize%22%3A%2225%22,%22SortBy%22%3A%22Title%22,%22SortOrder%22%3A%22ASC%22,%22SearchTerm%22%3A%22%22%7D,%22ItemsToCompare%22%3A%5B%5D,%22SelectedStandardIdList%22%3A%5B%5D%7D. Accessed 13 June 2016.

[27] Bilimoria KY, Raval MV, Bentrem DJ, et al. National assessment of melanoma care using formally developed quality indicators. J Clin Oncol. 2009;27(32):5445–51. 10.1200/JCO.2008.20.9965. Epub 2009 Oct 13. Erratum in: J Clin Oncol 2010 Feb 1; 28(4):708. 10.1200/JCO.2008.20.9965 19826131

[28] Del Turco MR, Ponti A, Bick U (2010). Quality indicators in breast cancer care. Eur J Cancer.

[29] The American Society of Breast Surgeons (2017). Quality Measures for CMS Quality Payment Programs. https://www.breastsurgeons.org/new_layout/programs/mastery/mips_2017.php. Accessed 25 May 2017.

[30] Whitacre E (2009). The Importance of measuring the measures. Ann Surg Oncol..

[31] Neuss MN, Malin JL, Chan S (2013). Measuring the improving quality of outpatient care in medical oncology practices in the United States. J Clin Oncol..

[32] Western Electric Company. Hawthorne Studies Collection, 1924–1961 (Inclusive): a finding aid. Baker Library, Harvard Business School. http://oasis.lib.harvard.edu//oasis/deliver/deepLink?_collection=oasis&uniqueId=bak00047. Accessed 9 Dec 2015.

[33] Tjoe JA, Greer DM, Ihde SE, Bares DA, Mikkelson WM, Weese JL (2015). Improving quality metric adherence to minimally invasive breast biopsy among surgeons within a multihospital health care system. J Am Coll Surg..

[34] Lied TR, Kazandjian VA (1990). A Hawthorne strategy: implications for performance measurement and improvement. Clin Perform Qual Health Care..

[35] The National Quality Strategy (2014) Using levers to achieve improved health and health care. http://www.ahrq.gov/workingforquality/reports/nqsleverfactsheet.htm. Accessed 13 June 2016.

[36] Quality Payment Program (2017) What’s the quality payment program? https://qpp.cms.gov/. Accessed 26 May 2017.

[37] Quality Payment Program (2017) The 2016 CMS Physician Quality Reporting System Qualified Clinical Data Registries. https://qpp.cms.gov/docs/QPP_QCDR_Self-Nomination_Fact_Sheet.pdf. Accessed 26 May 2017.

[38] Kiefe CI, Weissman NW, Allison JJ (1998). Identifying achievable benchmarks of care: concepts and methodology. Int J Qual Health Care.

[39] Hatfield MD, Ashton CM, Bass BL, Shirkey BA (2016). Surgeon-specific reports in general surgery: establishing benchmarks for peer comparison within a single hospital. J Am Coll Surg..

[40] Siddiqui NY, Tarr ME, Geller EJ (2016). Establishing benchmarks for minimum competence with dry lab robotic surgery drills. J Minim Invasive Gynecol..

[41] Jalili M, Hejri SM, Norcini JJ (2011). Comparison of two methods of standard setting: the performance of the three-level Angoff method. Med Educ..

[42] Hurtz G, Auerbach M (2003). A meta-analysis of the effects of modifications to the Angoff method on cutoff scores and judgment consensus. Educ Pscyhol Meas..

[43] Livingston SA, Zieky MJ (1982). Passing scores: a manual for setting standards of performance on educational and occupational tests. https://www.imca.org/sites/default/files/file_1862.pdf. Accessed 13 June 2016.

[44] Landercasper J, Bailey L, Clifford E (2017). The American Society of Breast Surgeons and quality payment programs: ranking, defining and benchmarking more than a million patient-quality measure encounters. Ann Surg Oncol..

[45] Centers for Medicare and Medicaid Services (2015) The Medicare access and CHIP reauthorization Act of 2015: Path to Value. https://www.cms.gov/medicare/quality-initiatives-patient-assessment-instruments/value-based-programs/macra-mips-and-apms/macra-lan-ppt.pdf. Accessed 26 May 2017.

[46] Congress.Gov (2015). Congressional Record H.R.2—Medicare Access and CHIP Reauthorization Act of 2015. https://www.congress.gov/bill/114th-congress/house-bill/2/text. Accessed 26 May 2017.

[47] Centers for Medicare and Medicaid Services (2016). 2016 Physician Quality Reporting System Qualified Clinical Data Registries. https://www.cms.gov/Medicare/Quality-Initiatives-Patient-Assessment-Instruments/PQRS/Downloads/2016QCDRPosting.pdf. Accessed 26 May 2017.

[48] Centers for Medicare and Medicaid Services (2017). MACRA Delivery System Reform, Medicare Payment Reform. https://www.cms.gov/medicare/quality-initiatives-patient-assessment-instruments/value-based-programs/macra-mips-and-apms/macra-mips-and-apms.html. Accessed 26 May 2017.

[49] Clarke-Pearson EM, Jacobson AF, Boolbol SK (2009). Quality assurance initiative at one institution for minimally invasive breast biopsy as the initial diagnostic technique. J Am Coll Surg..

[50] El-Tamer MB, Ward BM, Schifftner T, Neumayer L, Khuri S, Henderson W (2007). Morbidity and mortality following breast cancer surgery in women: national benchmarks for standards of care. Ann Surg..

[51] Al-Hilli Z, Thomsen KM, Habermann EB (2015). Reoperation for complications after lumpectomy and mastectomy for breast cancer from the 2012 National Surgical Quality Improvement Program (ACS-NSQIP). Ann Surg Oncol..

[52] Shahian DM, Grover FL, Prager RL (2015). The Society of Thoracic Surgeons voluntary public reporting initiative: the first 4 years. Ann Surg..

[53] van Dam PA, Tomatis M, Marotti L (2015). The effect of EUSOMA certification on quality of breast cancer care. Eur J Surg Oncol..

[54] National Quality Forum (2017) Measure Evaluation Criteria. https://www.qualityforum.org/Publications/2013/10/Review_and_Update_of_Guidance_for_Evaluating_Evidence_and_Measure_Testing_-_Technical_Report.aspx. Accessed 31 May 2017.

[55] The American Society of Breast Surgeons (2016) The certification and accreditation criteria of the American Society of Breast Surgeons for breast ultrasound and stereotactic biopsy. https://www.breastsurgeons.org/new_layout/programs/certification/index.php. Accessed 31 May 2017.

[56] Navathe AS, Emanuel EJ (2016). Physician peer comparisons as a nonfinancial strategy to improve the value of care. JAMA.

[57] American College of Surgeons (2017). ACS National Surgical Quality Improvement Program (ACS NSQIP). https://www.facs.org/quality-programs/acs-nsqip. Accessed 13 June 2016.

[58] Scott JW, Olufajo OA, Brat GA (2016). Use of national burden to define operative emergency general surgery. JAMA Surg..

[59] Landercasper J, Attai D, Atisha D (2015). Toolbox to reduce lumpectomy reoperations and improve cosmetic outcome in breast cancer patients: The American Society of Breast Surgeons Consensus Conference. Ann Surg Oncol..

[60] Landercasper J, Whitacre E, Degnim AC, Al-Hamadani M (2014). Reasons for re-excision after lumpectomy for breast cancer: insight from the American Society of Breast Surgeons Mastery^SM^ database. Ann Surg Oncol..

[61] Schulman AM, Mirrielees JA, Leverson G, Landercasper J, Greenberg C, Wilke LG (2017). Re-excision surgery for breast cancer: an analysis of the American Society of Breast Surgeons (ASBrS) Mastery^SM^ database following the SSO-ASTRO “no ink on tumor” guidelines. Ann Surg Oncol..

[62] The American Society of Breast Surgeons (2016). Sponsored patient registries for research. https://www.breastsurgeons.org/new_layout/programs/research.php. Accessed 31 May 2017.

[63] Hall BL, Huffman KM, Hamilton BH (2015). Profiling individual surgeon performance using information from a high-quality clinical registry: opportunities and limitations. J Am Coll Surg..

[64] Huffman KM, Cohen ME, Ko CY, Hall BL (2015). A comprehensive evaluation of statistical reliability in ACS NSQIP profiling models. Ann Surg..

[65] The American Society of Breast Surgeons (2016). Mission statement. https://www.breastsurgeons.org/new_layout/index.php. Accessed 22 Feb 2017.

